# Expectations of Tele-Yoga in Persons With Long-Term Illness: Qualitative Content Analysis

**DOI:** 10.2196/36808

**Published:** 2023-09-13

**Authors:** Towe Hedbom, Maria Liljeroos, Ingela Thylén, Lotti Orwelius, Tiny Jaarsma, Anna Strömberg

**Affiliations:** 1 Department of Health, Medicine and Caring Sciences Linkoping University Linkoping Sweden; 2 Centre for Clinical Research Sörmland Uppsala University Eskilstuna Sweden; 3 Department of Cardiology Linkoping University Linkoping Sweden; 4 Department of Anaesthesia and Intensive Care Linkoping University Linkoping Sweden; 5 Department of Clinical and Experimental Medicine Linkoping University Linkoping Sweden

**Keywords:** yoga, telerehabilitation, eHealth, chronic illness, heart failure, implantable cardioverter defibrillator, postintensive care

## Abstract

**Background:**

Yoga is a mind-body exercise that has demonstrated its feasibility and safety even for individuals with severe long-term illness. Engaging in yoga has the potential to yield positive effects on both physical and mental well-being. Tele-yoga is a novel approach to rehabilitation in which participants practice group yoga with a live-streamed yoga instructor digitally via a tablet. This is especially beneficial for individuals who may find it difficult to leave their homes to participate in an exercise session. As part of our ongoing evaluation of the tele-yoga intervention in individuals with long-term illness, we have undertaken an exploration of participants’ expectations regarding yoga in general and tele-yoga specifically. Understanding these expectations is crucial, as they can significantly impact their satisfaction with treatment and care and influence overall intervention outcomes.

**Objective:**

This study aims to explore the expectations of tele-yoga among individuals with long-term illness before starting a tele-yoga intervention.

**Methods:**

The study employed an inductive qualitative design and is part of a process evaluation within an ongoing randomized controlled trial. A total of 89 participants were interviewed before the start of the tele-yoga intervention. The interview guide encompassed questions about their general perceptions of yoga and the specific expectations they held for the upcoming tele-yoga sessions. The interviews were transcribed and analyzed using inductive qualitative content analysis.

**Results:**

Participants expressed their expectations for tele-yoga, focusing on the anticipated improvements in physical function and overall health. These expectations included hopes for reduced respiratory issues; relief from discomfort, aches, and pains; as well as increased physical flexibility, coordination, and overall well-being. Besides, they expected to achieve improved psychological well-being and performance; to acquire strategies to manage stress, anger, and anxiety; and to have their motivational drive strengthened and influence other activities. Participants described tele-yoga as a new and exciting technical solution that would facilitate the delivery of yoga. A few participants remained a little hesitant toward the use of technology, with some expectations based on previous experiences. When asked about expectations, some had no idea about what to expect. Participants also had varying perspectives on yoga, with some finding it mysterious and difficult to understand. Participants expressed thoughts that they found the idea of tele-yoga taking place in groups exciting and enjoyable. They also had expectations that being part of a group would provide opportunities for mutual inspiration and encouragement among the group members.

**Conclusions:**

Expectations before an intervention can provide valuable insights into understanding the factors influencing adherence to tele-yoga and its outcomes. Our findings provide a wide range of expectations for tele-yoga, spanning both physical and mental aspects. Moreover, the technology’s potential to facilitate yoga delivery and the supportive nature of digital group interactions were evident from the results.

**Trial Registration:**

ClinicalTrials.gov NCT03703609; https://clinicaltrials.gov/ct2/show/NCT03703609

## Introduction

### Background

Long-term illness is common and leads to limitations in daily life and to a decreased health-related quality of life in all dimensions among those affected [1-5]. A long-term illness is characterized by a condition that persists for 6 months or more. Such conditions encompass asthma, arthritis, cancer, kidney failure, diabetes, and heart disease [6].

Medical yoga is a therapeutic form of Kundalini yoga led by a certified yoga instructor that uses different standardized yoga programs with a combination of physical postures, breathing exercises, and relaxation/meditation specifically targeting individuals with long-term illness [7]. Medical yoga has been found to have several positive physiological effects on individuals with long-term illness, such as lowering blood pressure and heart rate [8-10]; managing pain [11]; and improving strength, endurance, balance, and other physical functions [12]. Previous studies have also found positive effects on health-related quality of life and reduced symptoms of depression for individuals living with heart failure when performing yoga [12,13]. Moreover, yoga has been found to assist individuals with paroxysmal atrial fibrillation, those with an implantable cardioverter defibrillator, and individuals dealing with cancer in relaxing and effectively managing their anxiety and fear [14-16].

People with long-term illnesses are often homebound and limited by fatigue, pain, and shortness of breath, which may be barriers to participation in center-based rehabilitation training [17,18]. The availability of the internet makes home-based training possible, even in group sessions [19]. Participants in a pilot study of tele-yoga intervention for persons with heart failure and chronic obstructive pulmonary disease reported enjoyment of yoga and appreciated the ability to attend a yoga class from home [20]. Tele-yoga offers the advantage of having no reported side effects or adverse events, making it a viable option with the potential for long-term positive effects [21-23].

Our research team is currently conducting a randomized controlled trial to evaluate the effects of tele-yoga in individuals with long-term illness. The design and pilot results of the randomized controlled trial have been previously published [22]. The tele-yoga intervention includes live-streamed yoga sessions for 1 hour, 2 times a week, at home via a videotelephony software program on a tablet. Each participant performs 20-24 yoga instructor–led group tele-yoga sessions over a period of 3 months. The intervention utilized medical yoga, which originates from Kundalini yoga. Medical yoga has been specifically developed to facilitate yoga for individuals with long-term medical conditions, such as heart failure. The medical yoga exercises can be performed while sitting on a chair, making them accessible and feasible to accommodate the needs and abilities of the participants. Two standardized yoga programs incorporating breathing exercises, physical postures, meditation, and relaxation are used. The tablet also includes an app providing instructions for yoga positions, breathing exercises, and meditation. Participants are encouraged to practice yoga at home individually using the app for a minimum of 50 minutes per week. This innovative approach enables rehabilitation remotely, which has become especially relevant during the COVID-19 pandemic. Digital rehabilitation increases the availability of rehabilitation for patients because they no longer need to travel to a facility [24]. Digital rehabilitation has been found to be not only effective and relevant [25] but also feasible and safe [26]. Previous studies have shown that on-site rehabilitation, compared with remote rehabilitation, yielded similar health-related quality of life outcomes. However, remote rehabilitation demonstrated more mental health benefits compared with in-person training [24].

Before engaging in digital rehabilitation, such as tele-yoga, the participants harbor expectations and perceptions about their upcoming experience. These expectations may sometimes be unformed and remain unexpressed. Expectations can stem from both cognitive and emotional aspects and are influenced by a person’s past experiences, knowledge, belief, hopes, needs, and external factors (eg, media, family, or friends) [27].

Expectations have been found to significantly impact satisfaction with treatment and care, and they also influence the outcomes of an intervention [28]. Understanding how expectations affect participants in a tele-yoga intervention can help identify and address barriers to and facilitators of adherence to the yoga training. Further, this understanding can provide insights into how expectations may influence the overall outcomes of the intervention [29].

### Aim

The objective of this study was to explore the expectations of individuals with long-term illness regarding a tele-yoga intervention before the actual initiation of the program.

## Methods

### Design and Sample

The study utilized a qualitative design with an inductive approach and was conducted as part of a process evaluation within a randomized controlled trial evaluating the effects of tele-yoga (ClinicalTrials.gov Identifier: NCT03703609). This study used a single-blind methodology where the evaluator and analyst were blinded, and it followed a parallel 2-arm randomized controlled study format. The participants were allocated in a 1:1 ratio to either the intervention group, which received tele-yoga, or the control group. A total of 200 individuals with long-term illness were recruited so far in the randomised controlled trial from 4 hospitals in southern Sweden and subsequently randomized into 2 groups. The first group (n=100) participated in a tele-yoga intervention conducted at home over a period of 3 months. The second group (n=100) served as the control group and received individual exercise advice during the same timeframe. The participants were recruited from those who had been admitted to the departments of cardiology or intensive care in the past 3-36 months, with a minimum length of stay of 48 hours during their hospitalization. To meet the training requirements, the participants were included in the study when they were stable in their medical condition and aged over 18 years. Exclusion criteria were short life expectancy (<6 months), impaired cognitive ability, lack of ability to fill in forms, and inability to complete the tele-yoga intervention. In this qualitative study, we consecutively invited the 100 patients randomized to the intervention group at baseline before starting the tele-yoga intervention to participate in an interview.

### Data Collection

We constructed a semistructured interview guide comprising a total of 10 questions (see Multimedia Appendix 1). Data were collected from October 2018 to September 2020. The interviews started with the opening question “Tell me what previous experiences you have of yoga.” Probing questions were asked to elucidate or let the respondent develop the answer further. The participants were interviewed either face-to-face or via telephone, depending on their preference or circumstances, during the COVID-19 pandemic. The interviews were conducted by the first author and skilled research nurses with previous experience in caring for this patient population. The interviewers had not met the participants before nor had any formal caring relationship with them. The first 2 interviews were considered pilot interviews and underwent meticulous scrutiny by the entire research team. The interview guide worked sufficiently well, so no changes were made after the pilot interviews and these interviews were therefore also included in the analysis. The length of the interviews varied between 3 and 18 minutes, with a mean of 8 minutes, and the interview material resulted in 300 A4 pages of transcribed text.

### Data Analysis

#### Overview

The interviews were tape-recorded and transcribed verbatim. Qualitative content analysis with an inductive approach was performed using the structure described by Elo and Kyngäs [30]. NVivo 12 Plus (QSR International) was used to facilitate the handling of a substantial amount of data, organize the data, and support the analysis.

#### Preparation Phase

Initially, the first 2 authors (TH and ML) independently read 20 transcribed interviews and identified units of analysis in the text that described different expectations of tele-yoga.

A meaningful unit comprised sentences and could encompass multiple meanings. Subsequently, the entire research team engaged in discussions to share their impressions of the text as a whole and the meaningful units identified.

#### Organizing Phase

In the next step, the first 2 authors (TH and ML) independently coded 5 randomly selected interviews, discussed their coding, and established a mutual agreement for the open coding. Following this agreement, they proceeded to analyze the remaining interviews. In the open coding process, notes and headings were written in the text while reading the interviews. The meaningful units were read and reread, and in the margins, as many headings as necessary were written to describe all aspects of the content. Discrepancies in coding were discussed until a consensus was reached. Meaningful units were then grouped under the respective headings. Each meaningful unit could be assigned to more than 1 code, and these codes were then organized and sorted on a coding sheet for further analysis. The codes were used to freely generate categories, which in turn were sorted and merged into fewer, more overarching categories. The first author maintained a log, in which the headings and ideas about relationships between categories were documented.

Throughout the analysis, the interviews were read repeatedly to gain an overall understanding of the full picture, and there was a constant comparison between the parts of the analysis and the raw data from the completed interviews.

During the abstraction phase, the structure and content of the categories and subcategories were discussed among all the authors.

### Ethical Consideration

The study was conducted in accordance with the ethical guidelines designed for studies of human research according to the World Medical Association Declaration of Helsinki [31]. Although human research studies can entail certain risks, the tele-yoga intervention in this study was assessed to have a low potential for causing injury or harm to participants. Studies involving human participants must uphold the fundamental principles of freedom and prioritize human welfare above the interests of research and societal needs [31]. The study participants received written and oral information about the study before signing informed consent. Participants were also informed that they had the option to withdraw their participation from the study at any time, and they were not required to provide a reason for doing so. The study was approved by the Ethical Review Board at Linkoping University Sweden (dnr 2017/225-31).

## Results

### Participants

The first 100 participants in the tele-yoga intervention group of the randomized controlled trial who were included between October 2018 and September 2020 were eligible for study inclusion and of these 89 consented to being interviewed. Among the 11 participants that did not participate, 2 declined due to lack of time and 9 were not invited due to organizational issues (eg, research staff vacation or difficulties reaching participants by phone to schedule the interview before the tele-yoga program started). As shown in Table 1, most participants (74/89, 83%) had no previous experience with yoga. A total of 10 participants had some previous experience practicing yoga or other similar activities such as chi gong, tai chi, mindfulness, and other types of relaxation exercises, while 5 had practiced yoga for several years. The participants’ ages ranged from 28 to 84 years, with 56/89 participants (63%) aged over 65 years. Two-thirds were men, and 82/89 (92%) study participants were born in Sweden; 3 out of 4 were in a relationship with someone and 71/89 (80%) had children. A total of 67 participants were diagnosed with heart failure (75%); additionally, 26 had diabetes mellitus, 13 had chronic obstructive pulmonary disease, 12 had been diagnosed with cancer, 12 had suffered a stroke, and 7 had renal failure. As many as 36 (40%) study participants had 1 chronic condition, 27 (30%) had 2 conditions, and the rest (26/89) had 3 or more (30%) chronic conditions, as measured by the Charlson Comorbidity Index [32].

**Table 1:** Demographic variables of the study participants (N=89).

Demographics			Value
Age (years), mean (SD)			64.8 (11.6)
**Gender, n (%)**			
	Men	60 (67)	
	Women	29 (33)	
**Living conditions^a^, n (%)**			
	Cohabiting	66 (75)	
	Living alone	22 (25)	
**Self-reported financial situation^a^, n (%)**			
	Very good finances	15 (17)	
	Good finances	63 (72)	
	Problematic finances	9 (10)	
	Very problematic finances	1 (1)	
**Primary occupation^a^, n (%)**			
	Working	26 (30)	
	Retired	53 (60)	
	Sick leave	4 (5)	
	Unemployed	2 (2)	
	Other	3 (3)	
**Educational level^a^, n (%)**			
	Primary school	17 (19)	
	Secondary school	6 (7)	
	Upper secondary school	32 (36)	
	College/university	33 (38)	
**Previous tablet use, n (%)**			
	Yes	48 (54)	
	No	41 (46)	
**Internet use amount, n (%)**			
	Almost every day	71 (80)	
	At least once a week, but not every day	7 (8)	
	Less than once a week	4 (5)	
	Never	7 (8)	

^a^Data missing for 1 study participant.

### Emerging Categories and Subcategories

During the interviews, we specifically inquired about the participants’ expectations regarding both the exercise format of the intervention (ie, medical yoga) and the delivery mode (ie, the technology to deliver the yoga; in this case, tele-yoga). Most participants had no prior experience with yoga or telerehabilitation. However, most participants described several diverse expectations about tele-yoga. Some participants, however, found it difficult to have expectations, because they either did not believe in yoga or had no idea what to expect.

**Figure 1:**
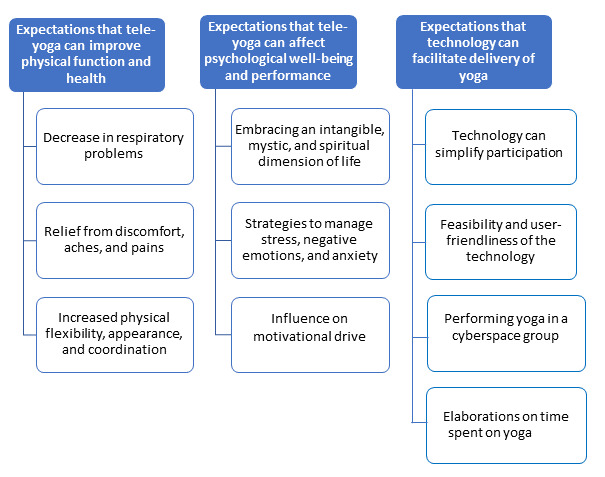
Categories and subcategories.

The analysis of the interviews resulted in the formulation of 3 categories and 10 subcategories (Figure 1).

### Expectations That Tele-Yoga Can Improve Physical Function and Health

#### Category Overview

This category encompasses the expectations of tele-yoga regarding the potential physical benefits that participants anticipated in yoga practice. The study participants were seeking ways to manage their symptoms, as they often felt a lack of control over their condition. They believed that tele-yoga could enhance their physical function and overall health.

The participants held various expectations regarding the improvement of physical function through tele-yoga. They envisioned benefits such as enhanced breathing, reduced pain, and improved flexibility and balance. Many of them experienced stiffness and frequent pain, and they believed that yoga could positively impact their daily life by addressing these issues.

#### Decrease in Respiratory Problems

Many participants described breathing difficulties, such as shortness of breath and breathlessness, as a result of their long-term illness. These symptoms significantly impacted their daily lives and overall well-being. The participants believed that tele-yoga would have a positive impact on their breathing and help reduce their respiratory problems in daily life. They hoped to learn techniques to better control their breathing and improve their overall respiratory function. They also described experiencing nocturnal respiratory problems that led to stress and expressed a desire to find techniques to manage these issues during the night. Although they did not often have a clear understanding of the specific breathing techniques in yoga, they perceived that it would be an important aspect of the tele-yoga sessions.

Since I have COPD, I sometimes have...Like last Sunday, I felt so bad, I couldn’t go from downstairs to upstairs without resting on the stairs halfway. I could have such difficulty breathing and get really short of breath and it is known that in yoga, you learn to breath.

participant 1-028, woman, 68 years

#### Relief From Discomfort, Aches, and Pain

The participants had expectations that tele-yoga training would provide relief from long-term pain and reduce aches and pains. They perceived tele-yoga as an alternative to painkillers, preferring to work with their body’s natural resources instead of solely relying on pharmacological solutions. Although they did not expect to be completely pain free, they hoped that tele-yoga could empower them to better manage and gain more control over their aches and pains. They perceived that the mind-body connection in yoga would help them overcome the influence of pain on their lives. Many described their pain as being difficult to manage and stated that stress aggravated the pain. They believed that tele-yoga could reduce stress and tension in the body and thus have a pain-relieving effect.

...to be completely pain-free, I know I will never be because I have such broken nerves eh...in my back. I know it will never be...//...I will be able to have a pain like...I couldn’t say that I accept it, but it must be there, it must be there on my terms. I am the one who decides how this pain should be allowed to act in my body, not the other way around. At the moment, it decides over me... and it’s very hard. Physically, but above all mentally. Because I couldn’t be the person I want to be, I fear I couldn’t be the father, for example, that I want to be to my children.

participant 1-039, man, 55 years

#### Increased Physical Flexibility, Appearance, and Coordination

Several participants had an expectation that tele-yoga would improve their body function and make them more active. Many had experienced reduced mobility and increased stiffness, and they believed that tele-yoga could address these issues by enhancing their flexibility and mobility. They anticipated that yoga postures would lead to a more flexible and supple body, enabling them to experience positive effects in various aspects of their lives. Stretching the body was perceived to be difficult, but effective, as it could lead to increased flexibility and mobility. For some participants, yoga seemed difficult to perform as it had physical challenges. This was especially the case when the yoga sessions required them to sit in complicated positions and the exercises involved bending and performing difficult body movements. Some expressed concerns about their ability to perform tele-yoga properly, especially if they lacked agility or flexibility.

Stiffness and inflexibility were also highlighted as areas of concern for performing yoga, as some had difficulty sitting on the floor. However, participants found the format of the tele-yoga intervention, performed on a chair, feasible.

Then I thought yoga, of course I’ve been a little afraid of it, because I’m stiff as an old goat and then I thought that it’s good anyway, to soften the body. Then, it can be fun to see if this can inspire me to get started, to run and exercise again.

participant 1-116, man, 66 years

### Expectations That Tele-Yoga Can Affect Psychological Well-Being and Performance

#### Category Overview

This category focused on the psychological aspects of tele-yoga, with participants expressing expectations of improved psychological well-being. They had both positive and negative thoughts about the spiritual dimensions of yoga. The participants expected that tele-yoga would help reduce stress and negative emotions such as anger, fear, anxiety, and passiveness through relaxation techniques. Additionally, they believed that tele-yoga would serve as a motivational driver for engaging in other forms of exercise and activities.

#### Embracing an Intangible, Mystic, and Spiritual Dimension of Life

Participants had varying perspectives on yoga, with some finding it mysterious and difficult to understand due to its roots in ancient Eastern religion and history. Some participants were more skeptical and judgmental, while some were more curious and open-minded about the practice.

....initially it came across as a bit fuzzy, I think, I have to say. But it’s an old oriental history, so it’s certainly useful to keep doing. Then we’ll see how it develops, we don’t know. I have no idea.

participant 1-020, man, 75 years

Some participants found the concept of yoga itself to be vague and unclear, struggling to fully comprehend all its aspects and the tele-yoga intervention. Some participants formed their expectations of yoga based on the experiences shared by their relatives and friends.

I’ve never tried it myself (yoga)... My wife does yoga but not me....I also want to try it.

participant 1-011, man, 73 years

Yes, it’s probably because of not having done any yoga personally, so you think it’s hocus-pocus, but it might work great on me, I have no idea. I think it’s worth a try anyway.

participant 1-015, man, 56 years

#### Strategies to Manage Stress, Negative Emotions, and Anxiety

Participants had expectations that tele-yoga would offer them relaxation and provide a sense of peace and tranquility in their daily life.

They also had expectations that tele-yoga would help reduce stress levels and improve stress management, leading to increased feelings of calmness and relaxation. They anticipated that tele-yoga could assist in reducing anger, regulating negative emotions, and enhancing their ability to cope with life’s challenges in a more positive and composed manner. During overwhelming moments, they hoped that tele-yoga could guide them back to a more balanced and relaxed state. By alleviating stress, they believed that it could also enhance their focus, inner peace, self-assurance, and overall well-being.

So partly I could be the kind of person who could be stressed inside and maybe not look so stressed on the outside. But inside I could stress myself out about things and I think maybe I could get some help with that. Calm down.

participant 1-082, woman, 53 years

Participants expressed expectations that tele-yoga could potentially alleviate anxiety through the relaxation and improved breathing techniques learned during the intervention. They also anticipated that tele-yoga might lead to improved sleep patterns, often associated with reduced stress and the relaxation benefits they believed it would offer. Additionally, they held expectations that tele-yoga could improve memory capacity. Furthermore, participants looked forward to experiencing an overall sense of well-being, positivity, and increased happiness through their participation in tele-yoga.

#### Influence on Motivational Drive

Participants anticipated that tele-yoga would have a positive impact on their overall physical activity levels.

I was not expecting to be part of that group but otherwise I had thought that it was something that would give me a kick in the butt to get started and move a little more again, it has been quite bad with that.

participant 1-054, man, 78 years

Some exercised regularly or were active in other ways, but thought that tele-yoga could offer them a different perspective on exercise. However, many participants did not exercise at all, but expected that tele-yoga could help motivate them to start exercising and help them to improve their physical fitness and lose weight. While their long-term illness had, in many cases, made them sedentary and inactive, some had problems moving and did not have any motivation to exercise.

Yes...it sounded interesting and might push me a little more...give me motivation to exercise more. Because that’s what is missing sometimes...the motivation.

participant 1-037, man, 78 years

### Expectations That the Technology Can Facilitate the Delivery of Yoga

#### Category Overview

This category encompasses participants’ expectations regarding the implementation of tele-yoga using technology. They expressed their thoughts about practicing yoga remotely in a group setting, as well as the time commitment required for the tele-yoga intervention during the study.

#### Technology Can Simplify Participation

Many participants were of the belief that the technology would facilitate participation in tele-yoga sessions from home and decrease traveling. They anticipated that performing tele-yoga would be comfortable and easy to follow from home.

I think it will be great not to have to go anywhere but to be at home in the environment where you are comfortable.

participant 1-061, woman, 49 years

Many participants recognized the flexibility that the technology offered, enabling them to participate in tele-yoga from various locations such as the workplace, summer house, or during travel. Several participants worked and some specifically mentioned planning to engage in yoga sessions from their workplace. They also appreciated how the technology simplified participation in tele-yoga and facilitated communication with both other participants and instructors.

Some were concerned about potential disruptions while participating in tele-yoga from home or work. They acknowledged that finding a suitable, quiet space to perform tele-yoga without disturbances or background noise was essential for a satisfactory experience.

#### Feasibility and User-Friendliness of the Technology

Participants’ expectations regarding the technology were influenced by their previous experiences with computers, tablets, smartphones, and other mobile devices. In general, most participants were familiar with the technology, described the use of technology as exciting, and did not consider it to be a problem. Even those who were not accustomed to using technology expressed their eagerness to use it and indicated that it was interesting to learn something new.

Some participants expressed concerns about using technology and shared negative perceptions and expectations. They mentioned feeling insecure and facing difficulties when using technology that they were not accustomed to. Besides, some had a strong need for control and therefore always found it difficult to manage new technology, as it was associated with a fear of making mistakes.

Some participants made more neutral statements, assuming that the technology would not be a problem but acknowledging that they would not know for sure until they had used it. Others mentioned that they had not given much thought about the technology aspect at all.

Despite some participants expressing a lack of confidence in the technology, they expected that they would gradually learn to use it. They understood that the technology was new and might require some effort to understand, but they were generally confident that they would learn to use it step by step. Additionally, some participants appreciated the convenience of a tablet, as it is easy to carry and use.

That it’s pretty simple, it feels like, it’s pretty good, I think since I have a tablet. Because it’s a larger screen than a phone and not as clumsy as a computer. I think it’s a good tool. Easy to bring with you if you happen to be somewhere else and so on. It feels good.

participant, 1-123, woman, 28 years

#### Performing Tele-Yoga in a Cyberspace Group

During the tele-yoga sessions, participants engaged in group meetings, and their expectations regarding group participation were explored. Some participants expressed disinterest or had negative thoughts about being part of a group. They mentioned being like a “lone wolf” or shared past negative experiences with group activities. Furthermore, some were concerned that creating a sense of group belonging might be challenging in an online setting compared with face-to-face interactions. It became evident that physical social togetherness was valued in the group, and there were concerns that this aspect might be lost during online sessions.

So, I think it’s fun to do things in a group. So, it’s the social interaction in everything...I’ll miss that now.

participant 1-002, man, 61 years

As the group sessions had not started yet, some participants had envisioned how tele-yoga and digital sessions would appear on their tablet screens. They wondered whether they would be able to see other participants or if only the yoga instructor would be visible. There were questions about whether it would truly feel like a group experience when meeting online and seeing each other only through the tablet screen. Some participants were not aware that they could communicate with other participants since the sessions involved videoconference meetings. On the other hand, some participants had not focused on the group aspect at all, being primarily concerned with their tele-yoga practice, making the group element seem less significant to them.

Most participants expressed positive expectations regarding the group. Participants expressed thoughts that they found the idea of tele-yoga taking place in groups exciting and fun. They also had expectations that being part of the group would provide opportunities for mutual inspiration and encouragement among the members. Some participants drew from their previous experiences in other types of group activities and acknowledged the benefits of being part of a group, such as receiving support to succeed in an activity. In addition, the opportunity to work with others and share thoughts and reflections on both the yoga and the disease was perceived as positive. Additionally, some participants had positive experiences with other types of group activities, such as online games.

#### Elaborations of Time Spent on Yoga

The participants were asked if they expected that the time allocated for the tele-yoga was reasonable and how much time they planned to spend doing yoga. The participants mostly answered that they would follow the allotted time and that it seemed appropriate, but some also felt that they might set aside more time, especially if tele-yoga had a clear effect after the sessions.

To begin with, it’s these two times, Tuesdays and Fridays, and we have to start with that. This is how you feel, if you think this makes sense, so you might spend more time, I don’t really know.

participant 1-008, man, 71 years

By contrast, others were more insecure, stating that they had difficulty specifying the specific time, as they were not sure how much time it would take.

However, a few stated more specifically how much time they planned to spend and gave suggestions varying from half an hour per day to 2 hours maximum.

Some participants expressed concerns about the time commitment required for tele-yoga, wondering whether they could dedicate such a significant amount of time to the practice. They questioned whether investing time in yoga would be worthwhile if they did not experience the desired benefits. On the other hand, some participants appreciated having scheduled sessions for tele-yoga, as it provided structure and a sense of accountability.

## Discussion

### Principal Findings

This study aimed to investigate the expectations of participants before a tele-yoga intervention, where the participants were expected to engage in both group and individual yoga training using technology. The main findings of the study revealed that several of the expectations regarding tele-yoga were normative expectations [27], which are characterized by being considered likely to occur. Several physiological and psychological outcomes that participants expressed as expectations, including positive effects on breathing, sleep, stress, improved health-related quality of life, and decreased pain and anxiety, have been demonstrated in previous studies as effects of yoga training [3,4,9-12,33].

Expectations that tele-yoga would result in significant weight loss, dramatic improvement in agility, and increased engagement in all other physical activities can, to some extent, be categorized as more ideal expectations, which include desires, visions, and hopes for what might happen [27].

According to Thompson and Suñol [27], predicted expectations are influenced by other factors, such as the media or others, as well as the participants’ prior knowledge, in this study specifically of yoga and technology. Expectations may also depend on other factors such as participants’ prior experience with yoga, the challenges/problems their illness poses, their age, and their perception of what yoga entails [34,35].

Some participants faced challenges in expressing their expectations regarding the tele-yoga intervention. Those who indicated being more hesitant and adopting a wait-and-see approach had what Thompson and Suñol [27] refer to as “unformed expectations.” Furthermore, those participating in the study based on their desire or upon the recommendations of others (eg, family members), as well as those who felt a sense of duty to participate when invited to contribute to research [36], also had unformed expectations [27].

High expectations have been associated with improved adherence to an intervention and positive outcomes, which correlates positively with symptom changes [37]. A study among male cancer survivors within the Veterans Health Administration showed that positive beliefs about yoga were associated with greater improvements in physical function from a yoga intervention [34]. In this study, participants who perceived tele-yoga as user-friendly and facilitating participation had a more positive attitude toward the use of technology. By contrast, participants who found the technology difficult and complicated and expressed concerns about its usage in general had an overall perception that they were less positive and had lower expectations. These findings confirm results from a previous systematic review and meta-synthesis [38], which showed that higher expectations increased the effects of cardiac rehabilitation.

Additionally, it became apparent that participants who were motivated to improve their physical and mental well-being displayed a more positive attitude toward yoga than those who participated for other reasons (eg, contributing to research and being able to help others or other external factors, such as their relatives wanting them to participate in the study). Some of our participants also encountered challenges in expressing their expectations. These participants lacked a clear understanding of yoga and were uncertain about what to expect from the intervention, leading to more negative or neutral expectations. Previous studies have indicated that a lack of self-motivation, vague exercise identity, and lower previous physical activity levels may reduce expectations [38]. These findings also emerged in our study. The participants’ long-term illness had physical implications that lead to reduced activity levels and many participants in this study had a sedentary lifestyle. Besides, many lacked the motivation to exercise.

Another study indicated that group yoga motivated their participants to become more active [39]. The tele-yoga group sessions might have motivated the participants to become more active and encouraged them to have a more active lifestyle with regular exercise, as several participants expressed this expectation.

Expectations play a crucial role in shaping individuals’ satisfaction with outcomes. In various fields, studies have shown that patients with high preoperative expectations face a higher risk of long-term dissatisfaction if those expectations are not met [40-42]. This emphasizes the significance of managing and aligning patients’ expectations before medical and rehabilitation interventions.

The COVID-19 pandemic posed challenges with rehabilitation, necessitating the need for remote rehabilitation [43,44]. Digital forms of rehabilitation can address the needs of persons with long-term illness. Moradi et al [45] concluded that telerehabilitation during a pandemic is an effective alternative for medical conditions such as stroke. Our study started before the COVID-19 pandemic and became even more relevant because the tele-yoga intervention could continue as planned despite the lockdown. The pandemic has underscored the significance of remote rehabilitation, and the successful adaptation of home-based activities suggests that they will likely remain a viable option in the future [46].

### Methodological Strength and Limitations

The consecutive selection is a strength as it ensures the inclusion of a representative sample of study participants. The substantial number of participants (N=89) has provided us with rich and representative data on expectations. As the question of expectations can be relatively broad, a larger sample is preferable. Regarding the scope of the interviews, some interviews were short, especially for the participants with mainly unformed expectations. Overall, the amount of data was sufficient and manageable, while not being too extensive.

All data were collected through interviews. The interviewers had no care relationship with the participants nor had ever met them before the interview, which created a sense of equality in power dynamics and facilitated open unbiased conversations. The interviews were primarily conducted via telephone, which meant body language, facial expressions, and reactions could not be captured. This limitation could have resulted in the interviewer missing opportunities to seek clarification for discrepancies between verbal and nonverbal communication. However, certain forms of response bias are reduced by the fact that the interviewer does not influence the participants through physical presence [47]. Telephone interviews are a common method that is accepted as reliable in qualitative studies [48,49]; particularly, during the COVID-19 pandemic there has been a strong emphasis on employing remote interviews. Telephone interviews have been recognized as particularly suitable for interviews that are expected to be more structured, focused, and shorter [50], which aligns well with the nature of our study. A recent study comparing video calls with face-to-face interviews did not find any difference in the length of the interviews [51]. The participants were initially allowed to choose between the 2 options. However, as a result of the COVID-19 pandemic, all interviews were later conducted via telephone.

To ensure trustworthiness, analyst triangulation was applied, with the researchers independently coding and categorizing data, and each step of the analysis process was discussed until consensus about the interpretation was achieved. A strength of the study is that the participants differed in age, gender, and other sociodemographic variables (eg, previous training experience), which provided a rich variety of information. All data in the interviews relevant to the aim have been included in the analysis. The data were very rich and sufficient to explore a broad range of expectations for tele-yoga.

### Conclusions

This study explored participants’ expectations before starting a tele-yoga intervention. By studying the expectations of the participants in the intervention, we can further understand what influences them to participate and how expectations can affect adherence to the tele-yoga intervention as well as the outcomes. Several expectations for tele-yoga emerged, including various aspects of improved physical function and health, along with increased mental well-being and performance. There was also an expectation that the technology could facilitate the delivery of yoga. This facilitates the understanding of what influences and motivates participants to participate in an intervention study focusing on digitally delivered yoga.

## Appendix

Multimedia Appendix 1 Interview guide.

## Data Availability

Data are stored according to the electronic data management plan at the Department of Health, Medicine and Caring Sciences at Linköping University.
