# Correction: Expectations of Tele-Yoga in Persons With Long-Term Illness: Qualitative Content Analysis

**DOI:** 10.2196/55077

**Published:** 2023-12-19

**Authors:** Towe Hedbom, Maria Liljeroos, Ingela Thylén, Lotti Orwelius, Tiny Jaarsma, Anna Strömberg

**Affiliations:** 1 Department of Health, Medicine and Caring Sciences Linkoping University Linkoping Sweden; 2 Centre for Clinical Research Sörmland Uppsala University Eskilstuna Sweden; 3 Department of Cardiology Linkoping University Linkoping Sweden; 4 Department of Anaesthesia and Intensive Care Linkoping University Linkoping Sweden; 5 Department of Clinical and Experimental Medicine Linkoping University Linkoping Sweden

In “Expectations of Tele-Yoga in Persons With Long-Term Illness: Qualitative Content Analysis” (J Med Internet Res 2023;25:e36808) the authors noted one omission.

The attached image will be added to the manuscript as Figure 1 at the end of the last paragraph within the “Emerging Categories and Subcategories” subheading, which originally appeared as:

The analysis of the interviews resulted in the formulation of 3 categories and 10 subcategories.

And will now read as follows:

The analysis of the interviews resulted in the formulation of 3 categories and 10 subcategories (Figure 1).

The correction will appear in the online version of the paper on the JMIR Publications website on December 20, 2023 together with the publication of this correction notice. Because this was made after submission to PubMed, PubMed Central, and other full-text repositories, the corrected article has also been resubmitted to those repositories.

**Figure 1:**
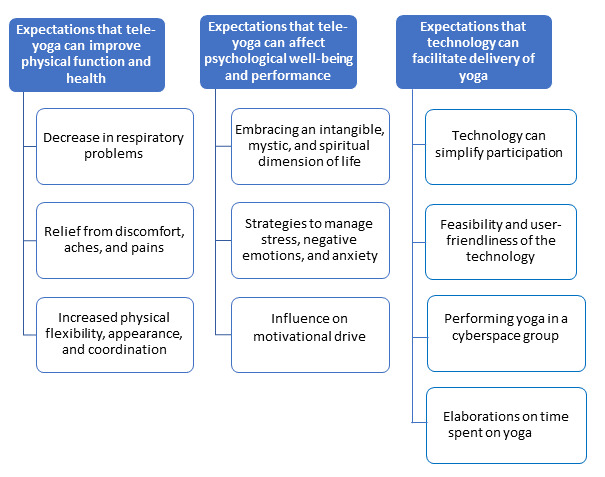
Categories and subcategories.

