# Supplementary material for: Expectations of Tele-Yoga in Persons With Long-Term Illness: Qualitative Content Analysis
Source: J Med Internet Res. 2023 Sep 13;25:e36808. doi: 10.2196/36808 (PMC10534282; doi:10.2196/36808)
Supplement: Multimedia Appendix 1 [file jmir_v25i1e36808_app1.docx]

**Appendix**

Interview guide
**___________________________________________________________________________**
Tell me what previous experiences you have of yoga? Have you encountered or had any experience of similar mindfulness methods such as breathing exercises, tai-chi or similar exercises?

Do you perform any type of exercise training or are you physically active?

Tell us what is motivating you to take part in this study?

What are your perceptions of yoga?

What effects do you think you can expect from the tele-yoga?

What do you think about the telecommunications technology that will be used in the app?

What do you think about the social aspect of being in an online group? In the yoga project you will do tele-yoga in a group, how do you feel about being in an online group?

How much time do you think it is reasonable for you to spend doing yoga (group yoga and using the app) each week?

Do you have any other thoughts or opinions regarding the study that you want to address now before the tele-yoga group starts?

Is there anything else you want to add?
